# Resveratrol Attenuates Microglial Activation via SIRT1-SOCS1 Pathway

**DOI:** 10.1155/2017/8791832

**Published:** 2017-07-11

**Authors:** Shuping Zhang, Lu Gao, Xiuying Liu, Tao Lu, Chuangbo Xie, Ji Jia

**Affiliations:** ^1^Department of Dermatology, The First Affiliated Hospital, Shantou University Medical College, Shantou 515041, China; ^2^Department of Neurosurgery, Xi'an Children's Hospital, Xi'an 710003, China; ^3^Guangzhou University of Chinese Medicine, Guangzhou 510045, China; ^4^Department of Anesthesiology, Guangzhou General Hospital of Guangzhou Military Command, Guangzhou 510010, China

## Abstract

Microglial activation is involved in a variety of neurological disorders, and overactivated microglial cells can secrete large amount of proinflammatory factors and induce neuron death. Therefore, reducing microglial activation is believed to be useful in treating the disorders. In this study, we used 10 ng/ml lipopolysaccharide plus 10 U/ml interferon *γ* (LPS/IFN*γ*) to induce N9 microglial activation and explored resveratrol- (RSV-) induced effects on microglial activation and the underlying mechanism. We found that LPS/IFN*γ* exposure for 24 h increased inducible nitric oxide synthase (iNOS) and nuclear factor *κ*B (NF-*κ*B) p65 subunit expressions in the cells and enhanced tumor necrosis factor *α* (TNF-*α*) and interleukin 1*β* (IL-1*β*) releases from the cells. RSV of 25 *μ*M reduced the iNOS and NF-*κ*B p65 subunit expressions and the proinflammatory factors' releases; the knockdown of silent information regulator factor 2-related enzyme 1 (SIRT1) or suppressor of cytokine signaling 1 (SOCS1) by using the small interfering RNA, however, significantly abolished the RSV-induced effects on iNOS and NF-*κ*B p65 subunit expressions and the proinflammatory factors' releases. These findings showed that microglial SIRT1-SOCS1 pathway may mediate the RSV-induced inhibition of microglial activation in the LPS/IFN*γ*-treated N9 microglia.

## 1. Introduction

Neuroinflammation is involved in a variety of neurological disorders, including brain ischemia, brain trauma, and neurodegenerative diseases [1–3]. As a vital immunocyte in the central nervous system (CNS), microglia can be activated in the neurological disorders above. Overactivation of microglia can enhance the inflammation in the CNS by secreting proinflammatory cytokines, leading to neuron death and neurological dysfunction ultimately [4, 5]. Therefore, inhibiting microglial activation is regarded as an effective therapy in treating the CNS diseases. At present, two main kinds of anti-inflammatory drugs are widely used clinically, steroidal anti-inflammatory drugs and nonsteroidal anti-inflammatory drugs (NSAIDs). Long-term use of steroidal anti-inflammatory drugs, such as dexamethasone and prednisone, may induce immune dysfunction, infection, and lipid metabolism disorders [6, 7]. In contrast, administration of NSAIDs, such as aspirin, ibuprofen, and indometacin, may cause gastric ulcer, coagulation disorders, and renal dysfunction [8–10]. Given these reasons, searching for effective anti-inflammatory drugs with fewer and more mild side effects is a vital issue in treating inflammatory disorders.

Resveratrol is a bioactive substance rich in grape and Asian herbal medicine* Polygonum cuspidatum* [11, 12]. Some latest investigations showed that resveratrol can reduce microglial activation in brain ischemia and neurodegenerative disorders [13, 14]. And some studies showed that resveratrol can reduce brain injury in brain ischemia and Alzheimer's disease (AD) by increasing silent information regulator factor 2-related enzyme 1 (SIRT1) [15, 16]. However, the accurate mechanism of resveratrol in reducing microglial activation is still elusive. Suppressor of cytokine signaling 1 (SOCS1) is a protein, expressed in lymphocytes and microglial cells [17, 18]. Overexpression of SOCS1 protein decreases the activation of macrophage in the peripheral tissue and microglia in the CNS [19, 20].

In this study, we used lipopolysaccharide (LPS) plus interferon gamma (IFN*γ*), two proinflammatory substances, to activate N9 microglial cells [21], and took resveratrol to decrease the activation of the cells. We hypothesized that resveratrol can decrease the activation of N9 microglial cells exposed to LPS plus IFN*γ*, and microglial SIRT1-SOCS1 pathway may mediate the anti-inflammatory process of resveratrol.

## 2. Materials and Methods

### 2.1. Materials

N9 cell, a mouse microglial cell line, was obtained from the Fourth Military Medical University, China. The N9 microglial cells are very similar to the primary cultured microglial cells in producing cytokines and reacting to stimulus. The IMDM cell culture medium, fetal bovine serum (FBS), LPS (*Escherichia coli*, O55:B5), IFN*γ*, methylthiazol tetrazolium (MTT), and resveratrol were purchased from Sigma-Aldrich (USA). The SIRT1-siRNA, SOCS1-siRNA, and scrambled- (SC-) siRNA were purchased from Santa Cruz Biotechnology (USA). Antibodies against iNOS, SIRT1, SOCS1, and NF-*κ*B p65 subunit were purchased from the Abcam (Cambridge, UK). Antibodies against *β*-actin and GAPDH and Cy3-labeled secondary antibody were obtained from the Beijing Comwin Biotech Co., Ltd. (China).

### 2.2. Cell Culture and Treatments

The N9 microglial cells were cultured in the IMDM medium, containing 5% FBS, 100 U/ml penicillin, 100 *μ*g/ml streptomycin, and 2 mM glutamine. The air in the cell incubator was humidified and contained 5% CO_2_ and 95% air at 37°C. The medium was changed every 3 days. The stock cells were passaged 2-3 times per week with a split ratio of 1 : 4, and the cells were used within 8 weeks.

To find a suitable resveratrol (RSV) treatment concentration, the microglial cells were divided into five groups, including the LPS/IFN*γ* group, cells cultured in the medium containing 10 ng/ml LPS and 10 U/ml IFN*γ*, and four RSV treatment groups, cells cultured in the medium containing different concentrations of RSV (5, 10, 25, and 50 *μ*M), 10 ng/ml LPS, and 10 U/ml IFN*γ*. After 24 h incubation, western blot was used to evaluate iNOS protein expression in the cells. Then, to explore the role of SIRT1 in RSV-induced effects on microglial activation, we used SIRT1-siRNA to knock down the SIRT1 protein expression. The cells were divided into three groups: control: cells were cultured in the serum-free medium for 6 h and then exposed to normal medium for 6 h; SIRT1-siRNA: cells were cultured in the serum-free medium containing 90 pmol SIRT1-siRNA for 6 h; scrambled- (SC-) siRNA: cells were cultured in the serum-free medium containing 90 pmol SC-siRNA for 6 h and then exposed to normal medium for 6 h, and western blot was used to assess SIRT1 expression level. Then, the cells were divided into five groups: control group, LPS/IFN*γ* treatment group, 25 *μ*M resveratrol (RSV) + LPS/IFN*γ* group, SIRT1-siRNA + RSV + LPS/IFN*γ* group, and SC-siRNA + RSV + LPS/IFN*γ* group. After the treatments, western blot was performed to assess the iNOS and SOCS1 protein expressions, enzyme-linked immunosorbent assay (ELISA) was used to measure TNF-*α* and IL-1*β* concentrations in the medium, and immunocytochemistry was taken to observe iNOS expression. Furthermore, in order to explore the role of SOCS1 in resveratrol-induced effects, we took SOCS1-siRNA to silence SOCS1 protein expression. The cells were divided into three groups: control: cells were cultured in the serum-free medium for 6 h and then exposed to normal medium for 6 h; SOCS1-siRNA: cells were cultured in the serum-free medium containing 60 pmol SOCS1-siRNA for 6 h; SC-siRNA: cells were cultured in the serum-free medium containing 60 pmol SC-siRNA for 6 h and then exposed to normal medium for 6 h, and western blot was used to determine the SOCS1 expression. The cells were then divided into five groups: control group, LPS/IFN*γ* treatment group, RSV + LPS/IFN*γ* treatment group, SOCS1-siRNA + RSV + LPS/IFN*γ* group, and SC-siRNA + RSV + LPS/IFN*γ* group. After the treatments, western blot was performed to assess the iNOS and NF-*κ*B p65 subunit expressions and ELISA was used to measure TNF-*α* and IL-1*β* concentrations in the medium.

### 2.3. Cell Viability Assay

The cells were seeded in a 96-well cell culture plate at a density of 1 × 10^4^ cells/well. After the treatments, 20 *μ*l of MTT solution (5 mg/ml) was added to each well. After 4 h incubation at 37°C, the medium in the 96-well plate was removed, and 150 *μ*l of dimethyl sulfoxide (DMSO) was added to each well to dissolve the formazan product. Then, the plate was shaken for 10 min to make all the formazan dissolve completely. The absorbance was then detected at 490 nm wavelength by using a spectrophotometer (TECAN, CH). The values were expressed as percentage of the control group.

### 2.4. Western Blot Analysis

The microglial cells were seeded in 6-well cell culture plates at a density of 2 × 10^6^ cells/well. After the treatments, the cells were collected. Then, the total protein was evaluated by using the Bradford method. The western blot analysis was performed as described previously [22]. The following primary antibodies against iNOS (1 : 1000), SIRT1 (1 : 1000), SOCS1 (1 : 1000), NF-*κ*B p65 subunit (1 : 1000), GAPDH (1 : 1000), and *β*-actin (1 : 1000) were used in this study. Chemiluminescence technique was taken to detect the antigens. Image analysis was performed by using computerized analysis software from Bio-Rad Laboratories (USA).

### 2.5. ELISA

The microglial cells were plated in 24-well cell culture plates at a density of 1 × 10^5^ cells/well, and, after the treatments, TNF-*α* and IL-1*β* concentrations in the supernatants were measured by using the corresponding Reagent Kit (Nanjing Jiancheng Bioengineering Institute, China). Briefly, after the treatments, the supernatants from the cell culture medium were collected by centrifugation at 14000 rpm for 5 min at room temperature. Then the levels of TNF-*α* and IL-1*β* were tested according to the manufacturer's instructions.

### 2.6. Immunocytochemistry

The microglial cells were seeded in confocal microscope specific cell culture plates at a density of 5 × 10^4^ cells/well. After the treatments, the cells were fixed with 4% paraformaldehyde for 30 min at room temperature. Then the cells were washed three times with phosphate buffered saline (PBS). Next, the cells were exposed to 5 mg/ml bovine serum albumin for 30 min, followed by three times of washing with PBS. The cells were incubated in anti-iNOS primary antibody (1 : 50 in dilution) overnight at 4°C. After the incubation, the cells were washed with PBS three times, 5 min per time. Then the cells were incubated in Cy3-labeled secondary antibody (1 : 100 in dilution); after 1 h incubation at room temperature, 200 *μ*l of DAPI solution was added to each cell culture plate. After 10 min incubation in dark at room temperature, the plates were washed with PBS three times, 5 min per time. Then the cells were observed by using the confocal microscope (Olympus, Japan); photos were taken randomly.

### 2.7. siRNA Interfering

To downregulate SIRT1 or SOCS1 protein expression in the N9 microglial cells, the cells were treated with 90 pmol SIRT1-siRNA or 60 pmol SOCS1-siRNA by using the Lipofectamine reagent (Invitrogen, USA) in serum-free medium, according to the manufacturer's instructions. The cells were incubated for 6 h and recovered for an additional 6 h before the exposures of the drugs. The SC-siRNA was taken as the negative control.

### 2.8. Statistical Analysis

In this study, SPSS 13.0 for Windows (SPSS Inc., USA) was taken to conduct the statistical analysis. All the values of this study were expressed with means ± standard deviation (SD). The results of the groups were compared by one-way ANOVA, followed by Tukey's Multiple Comparison Test. *P* < 0.05 indicates statistical significance.

## 3. Results

### 3.1. Resveratrol Reduced iNOS Expression and Restored Cell Viability in Microglial Cells Exposed to LPS/IFN*γ*

**Figure 1:**
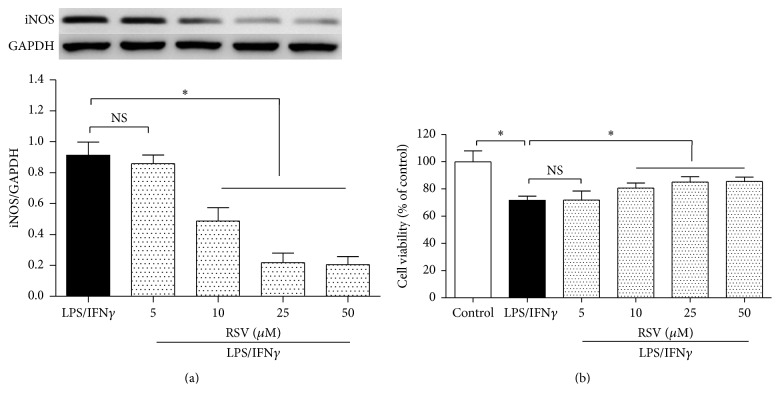
Resveratrol reduced iNOS expression and restored cell viability in the microglial cells exposed to LPS plus IFN*γ*. (a) The N9 microglial cells were divided into five groups, including 10 ng/ml LPS plus 10 U/ml IFN*γ* (LPS/IFN*γ*) exposure group, and four concentrations of resveratrol (RSV) treatment groups (5 *μ*M, 10 *μ*M, 25 *μ*M, and 50 *μ*M). After 24 h incubation, western blot analysis was taken to evaluate the iNOS protein expression in the cells (*n* = 4). (b) The cells were divided into six groups, including control, LPS/IFN*γ*, and four concentrations of resveratrol (RSV) treatment groups (5 *μ*M, 10 *μ*M, 25 *μ*M, and 50 *μ*M). After 24 h incubation, MTT assay was taken to evaluate the cell viability (*n* = 8). Results are expressed as means ± SD. ^*∗*^*P* < 0.05; NS: no significance.

To activate N9 microglial cells and mimic neuroinflammation, we used 10 ng/ml LPS plus 10 U/ml IFN*γ* and took western blot analysis and MTT assay to evaluate iNOS protein expression and cell viability level, respectively. A high iNOS expression level indicates microglial activation. The N9 microglial cells were divided into five groups (Figure 1(a)): LPS/IFN*γ* exposure group and 5 *μ*M, 10 *μ*M, 25 *μ*M, and 50 *μ*M of resveratrol treatment groups. After 24 h treatment, compared with the LPS/IFN*γ* exposure group, 10 *μ*M, 25 *μ*M, and 50 *μ*M of resveratrol reduced the microglial iNOS expression significantly (*P* < 0.05). Then, the cells were divided into six groups, control group and the five groups as above (Figure 1(b)). After 24 h treatment, LPS/IFN*γ* exposure reduced the cell viability significantly (*P* < 0.05), and 10 *μ*M, 25 *μ*M, and 50 *μ*M of resveratrol restored the cells viability markedly (*P* < 0.05). These findings showed that resveratrol exposure can reduce microglial activation and injury in the presence of LPS/IFN*γ*. And 25 *μ*M of resveratrol was used in the subsequent experiments to explore the anti-inflammatory effects of the drug.

### 3.2. SIRT1-siRNA Reversed Resveratrol-Induced Inhibition of Microglial Activation Significantly

**Figure 2:**
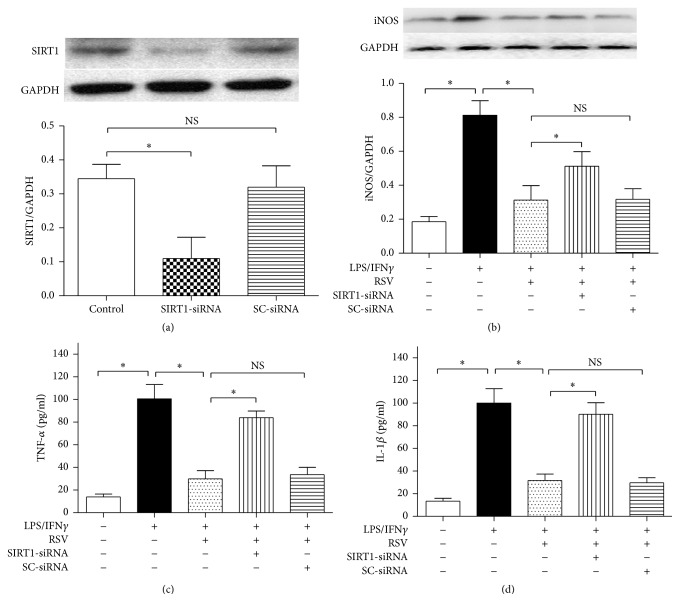
SIRT1-siRNA reversed resveratrol- (RSV-) induced effects on iNOS expression and proinflammatory factors' releases. (a) SIRT1-siRNA was effective in downregulating SIRT1 protein expression. The cells were divided into three groups as shown in the figure. After 6 h incubation, the SIRT1 protein expression was assessed by western blot analysis (*n* = 4). (b) Microglial iNOS protein expression (*n* = 4). (c) TNF-*α* concentration in the supernatants (*n* = 8). (d) IL-1*β* concentration in the supernatants (*n* = 8). The cells were divided into five groups and treated with different drugs as shown in the figure. After 24 h incubation, western blot and ELISA kits were used to assess iNOS expression and proinflammatory factors' releases, respectively. Results are expressed as means ± SD. ^*∗*^*P* < 0.05; NS: no significance.

**Figure 3:**
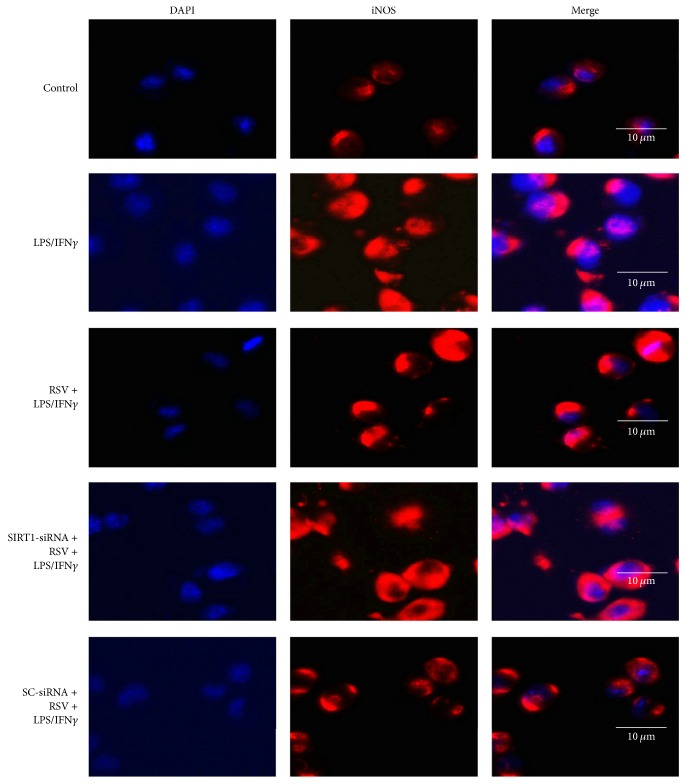
SIRT1-siRNA reversed resveratrol- (RSV-) induced effects on iNOS expression. The cells were divided into five groups and treated with different drugs as shown in the figure. After 24 h incubation, immunocytochemistry was used to observe the iNOS expression (red) in microglial cells, and the nuclei (blue) were counterstained with DAPI staining solution. Bar = 10 *μ*m.

To explore the role of SIRT1 in resveratrol-induced effects on microglial activation, we used SIRT1-siRNA. We found that SIRT1-siRNA downregulated SIRT1 protein expression in N9 microglial cells markedly (*P* < 0.05), but the scrambled siRNA (SC-siRNA) did not (Figure 2(a)). Then we explored the role of SIRT1 in resveratrol-induced effects on iNOS expression (Figures 2(b) and 3). Compared with the cells exposed to 10 ng/ml LPS plus 10 U/ml IFN*γ*, 25 *μ*M of resveratrol reduced iNOS protein expression obviously (*P* < 0.05); the effect, however, was significantly reversed by SIRT1-siRNA. The SC-siRNA did not cause significant effect on iNOS expression, compared with the resveratrol treatment group.

We also assessed the concentrations of TNF-*α* and IL-1*β*, two proinflammatory factors, in the supernatants of the cell culture medium (Figures 2(c)-2(d)). Compared with the cells cultured in the drug-free medium, LPS/IFN*γ* exposure for 24 h increased the TNF-*α* and IL-1*β* concentrations (*P* < 0.05), and 25 *μ*M resveratrol significantly reduced the two cytokines' levels (*P* < 0.05); the SIRT1-siRNA, not the SC-siRNA, however, partially abolished the resveratrol-induced effects on TNF-*α* and IL-1*β* levels (*P* < 0.05). These findings above showed that SIRT1 may mediate resveratrol-induced inhibition of microglial activation in the N9 microglial cells exposed to LPS/IFN*γ*.

### 3.3. SIRT1-siRNA Reversed Resveratrol-Induced Effects on SOCS1 Protein Expression in Microglial Cells

**Figure 4:**
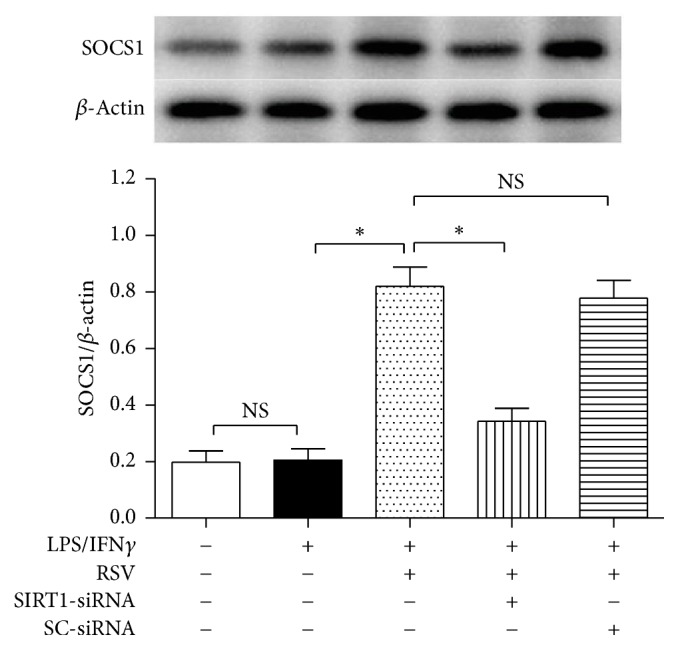
SIRT1-siRNA reversed resveratrol- (RSV-) induced effects on SOCS1 expression. The cells were divided into five groups and treated with different drugs as shown in the figure. After 24 h incubation, western blot was used to evaluate the SOCS1 protein expression in microglial cells (*n* = 4). Results are expressed as means ± SD. ^*∗*^*P* < 0.05; NS: no significance.

To observe the interaction between SOCS1 and SIRT1 proteins, we used western blot to evaluate the SOCS1 protein expression in the presence of SIRT1-siRNA and resveratrol (Figure 4). Compared with the cells exposed to 10 ng/ml LPS plus 10 U/ml IFN*γ* alone, 25 *μ*M resveratrol increased the SOCS1 expression level significantly (*P* < 0.05); however, the SIRT1-siRNA, not the SC-siRNA, abolished the upregulation of SOCS1 protein expression caused by resveratrol obviously (*P* < 0.05).

### 3.4. SOCS1-siRNA Reversed Resveratrol-Induced Inhibition of Microglial Activation

**Figure 5:**
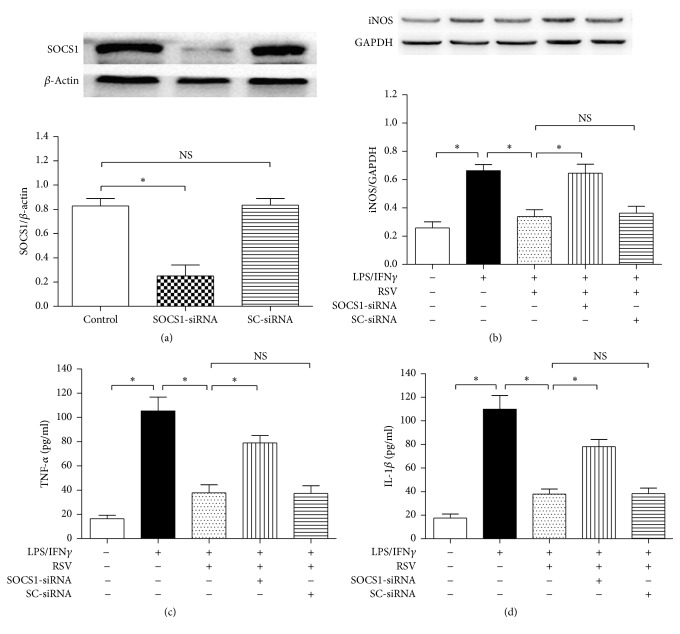
SOCS1-siRNA reversed resveratrol- (RSV-) induced effects on iNOS expression and proinflammatory factors' releases. (a) SOCS1-siRNA was effective in reducing SOCS1 protein expression in N9 microglial cells. The N9 microglial cells were divided into three groups as shown in the figure. After 6 h incubation, the SOCS1 protein expression was assessed by western blot analysis (*n* = 4). (b) Microglial iNOS protein expression (*n* = 4). (c) TNF-*α* concentration in the supernatants (*n* = 8). (d) IL-1*β* concentration in the supernatants (*n* = 8). The cells were divided into five groups and treated with different drugs as shown in the figure. After 24 h incubation, western blot and ELISA kits were used to assess iNOS expression and proinflammatory factors' releases, respectively. Results are expressed as means ± SD. ^*∗*^*P* < 0.05; NS: no significance.

To observe the role of SOCS1 in resveratrol-induced inhibition of microglial activation, we took SOCS1-siRNA to knock down the SOCS1 protein expression. In this experiment (Figure 5(a)), the SOCS1-siRNA reduced the SOCS1 protein expression significantly (*P* < 0.05). Then, by using western blot analysis, we measured the iNOS protein expression (Figure 5(b)). Compared with the cells cultured in drug-free medium, 10 ng/ml LPS plus 10 U/ml IFN*γ* exposure for 24 h upregulated the iNOS protein expression in the N9 microglial cells, and coadministration of 25 *μ*M resveratrol reduced the iNOS expression markedly in the presence of LPS/IFN*γ*; SOCS1-siRNA, not SC-siRNA, however, significantly reversed the resveratrol-induced reduction of iNOS expression (*P* < 0.05).

Similarly, compared with the cells treated with LPS/IFN*γ*, 25 *μ*M resveratrol decreased the TNF-*α* and IL-1*β* concentrations in the supernatants of the medium (*P* < 0.05), but the SOCS1-siRNA significantly reversed the resveratrol-induced effects on the levels of the two cytokines (*P* < 0.05, Figures 5(c)-5(d)), and the SC-siRNA did not cause significant influence on the levels of the two cytokines in the supernatants of the medium (*P* > 0.05).

### 3.5. SOCS1-siRNA Reversed Resveratrol-Induced Reduction of NF-*κ*B p65 Subunit Expression in the Microglial Cells

**Figure 6:**
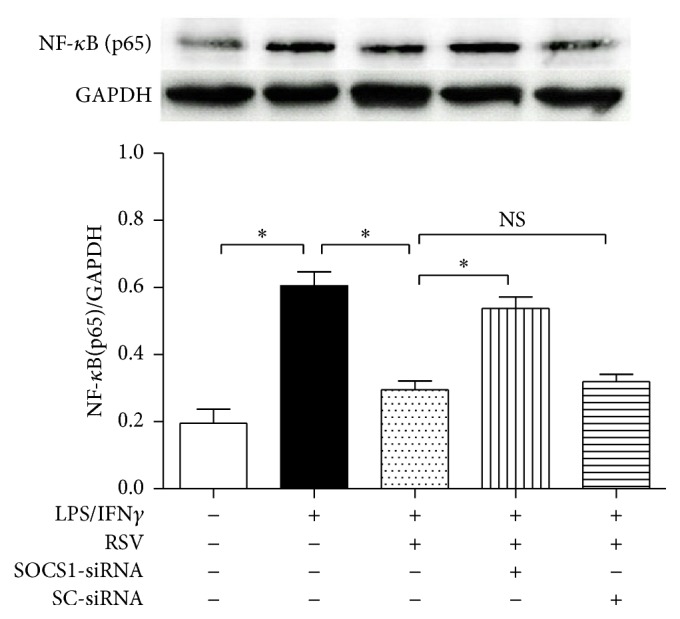
SOCS1-siRNA reversed resveratrol- (RSV-) induced effects on NF-*κ*B p65 subunit expression. The cells were divided into five groups and treated with the drugs as shown in the figure. After 24 h incubation, western blot was used to assess the protein expression of NF-*κ*B p65 subunit (*n* = 4). Results are expressed as means ± SD. ^*∗*^*P* < 0.05; NS: no significance.

NF-*κ*B family consists of at least five members, including p65 (RelA), RelB, c-Rel, p50/105 (NF-*κ*B1), and p52/100 (NF-*κ*B2). NF-*κ*B p65 subunit can translocate from cytoplasm to the nucleus and bind inflammation-associated genes. Therefore, a high expression of NF-*κ*B p65 subunit indicates an enhanced inflammation and activation degree in microglial cells [23]. In this study, by using western blot (Figure 6), compared with the cells cultured in drug-free medium, we found that 10 ng/ml LPS plus 10 U/ml IFN*γ* exposure for 24 h increased the NF-*κ*B p65 subunit expression (*P* < 0.05), and 25 *μ*M resveratrol reduced the NF-*κ*B p65 subunit expression obviously; however, the SOCS1-siRNA partially abolished the resveratrol-induced effect on NF-*κ*B p65 subunit expression. These findings above showed that the SOCS1 protein may be involved in resveratrol-induced inhibition of microglial activation.

## 4. Discussion

In this study, we demonstrated that 10 ng/ml LPS plus 10 U/ml IFN*γ* exposure for 24 h increased the protein expressions of iNOS and NF-*κ*B p65 subunit in the N9 microglial cells and enhanced TNF-*α* and IL-1*β* concentrations in the supernatants of the medium, and coadministration of 25 *μ*M resveratrol decreased iNOS and NF-*κ*B p65 subunit protein expressions and reduced the two cytokines' levels. SIRT1-siRNA or SOCS1-siRNA, not the SC-siRNA, however, significantly reversed the resveratrol-induced effects above. These findings indicated that resveratrol attenuates microglial activation induced by LPS plus IFN*γ*, and microglial SIRT1-SOCS1 pathway may mediate the resveratrol-induced anti-inflammatory effects.

Resveratrol was first isolated from the roots of white hellebore and was named by Dr. Michio Takaoka in 1940 [24]. In 1963, resveratrol was isolated from* Polygonum cuspidatum*, a traditional Chinese and Japanese medicine [25]. At present, resveratrol has been found in skin and seeds of over 70 plants, including grape, berries, grains, tea, and peanuts [26, 27]. In recent years, a large number of studies were performed to investigate the potential medical applications of resveratrol, including antitumor, anti-inflammation, and antioxidation applications and preventing cardiovascular disorders [28–30]. As red wine is rich in resveratrol, this is believed to be the essential factor in the French Paradox [31], a term used frequently to summarize the epidemiological observation that the French have a very low incidence of coronary heart disease despite having a diet rich in saturated fats. In addition, some latest investigations showed that resveratrol induces neuroprotections; the exact mechanism, however, is still elusive. As many studies indicated that resveratrol is a potent agonist for SIRT1, and SIRT1 upregulation induces neuroprotective and anti-inflammatory effects [15, 16]; therefore, we investigated the role of SIRT1 in resveratrol-induced effects in microglial cells exposed to LPS plus IFN*γ*. We found that LPS plus IFN*γ* increased iNOS expression and proinflammatory cytokines' levels in the cell culture medium and resveratrol reduced the iNOS and the cytokines' levels, indicating that LPS/IFN*γ* activated microglial cells and resveratrol caused inhibition of the microglial activation. As the overexpression of SOCS1 protein induces anti-inflammatory effects in microglia and macrophages, and SIRT1 overexpression leads to the methylation of SOCS1 protein [32]; therefore, we also observed the role of SOCS1 in this study. Interestingly, we found that resveratrol exposure increased the SOCS1 protein expression and SIRT1-siRNA partially abolished the SOCS1 protein expression in microglial cells; this result revealed that the SOCS1 protein might be involved in the resveratrol-induced anti-inflammation in the current study. Then we investigated whether the SOCS1 protein mediates the resveratrol-induced effects. Similarly, the SOCS1-siRNA significantly reversed the resveratrol-induced effects on microglial activation and NF-*κ*B p65 subunit expression, indicating that the SOCS1 protein may mediate the resveratrol-induced anti-inflammation in LPS/IFN*γ*-treated microglial cells. In fact, the SOCS protein family consists of at least eight proteins, including SOCS1–7 and cytokine-inducible SH2 protein (CIS) [33–35]. All the SOCS proteins share a central SRC-homology 2 (SH2) domain, a variable N-terminal region containing an extended SH2 subdomain and a conserved SOCS box at the C-terminus [36]. However, there are closer structures in pairs among the SOCS proteins family: SOCS1 and SOCS3, SOCS4 and SOCS5, SOCS6 and SOCS7, and SOCS2 and CIS. Being different from other SOCS proteins, SOCS1 and SOCS3 are expressed in microglial cells, and some latest studies showed that SOCS1 overexpression can reduce microglial activation by shifting microglial M1 state to M2 state [36]. M1 state of microglial can enhance the secretion of proinflammatory cytokines from the cells, which is believed to be harmful. In contrast, microglia of M2 state can release anti-inflammatory cytokines and neurotrophic factors, including IL-10, glial cell derived neurotrophic factor (GDNF), and brain derived neurotrophic factor (BDNF); therefore, M2 state is neuroprotective. Although we did not test the biomarkers of microglial M2 state, we infer that resveratrol can inhibit LPS/IFN*γ*-induced microglial activation via the SIRT1-SOCS1 pathway and the inhibition of microglial activation may be associated with the modulation of microglial activated states. Similarly, Dragone et al. found that resveratrol exposure decreased LPS-induced murine N13 microglial activation cells by increasing the SOCS1 signaling pathway [37]. Moreover, in a 1-methyl-4-phenyl-1,2,3,6-tetrahydropyridine- (MPTP-) induced Parkinson's disease (PD) mouse model, Lofrumento et al. revealed that resveratrol treatment significantly reduced glial activation and decreased the levels of IL-1*β*, IL-6, and TNF-*α* in the brain tissue by upregulating the SOCS1 protein expression [38]. Additionally, Ma et al. reported that resveratrol decreased the immune response of LPS-stimulated RAW264.7 macrophages via the SOCS1 pathway [39]. As microglial cells are regarded as the macrophages in the brain, these findings above are in accordance with that of ours to a large extent.

However, there are still some limitations in our investigation; first, in this study, we just observed the microglial activation in a microglial cell line, not in primary cultured microglial cells and in vivo. Therefore, the findings of this study should be verified in vivo and in primary cultured cells. Second, SOCS3 is also expressed in microglial cells; whether SOCS3 is involved in the resveratrol-induced anti-inflammatory effects is still under investigation. These questions will be answered in our coming work.

In summary, according to the findings of this investigation, resveratrol attenuates LPS plus IFN*γ*-induced microglial activation, and the SIRT1-SOCS1 pathway may mediate the anti-inflammation of resveratrol in the N9 microglial cells.
